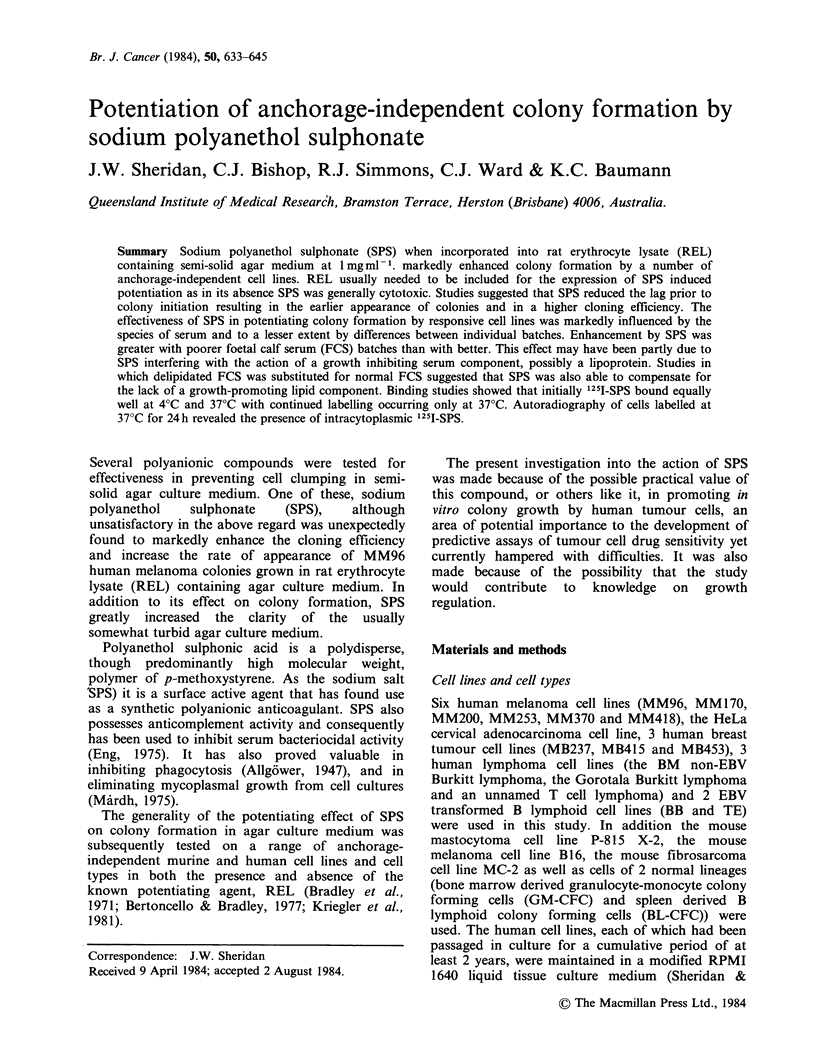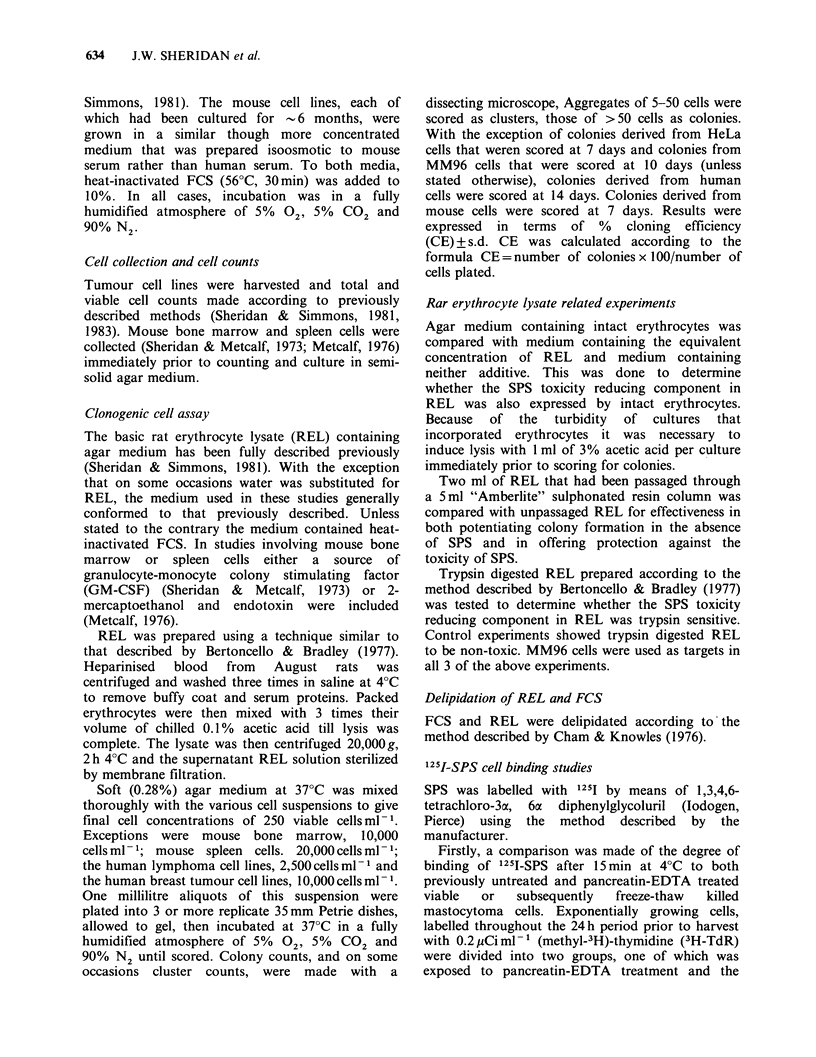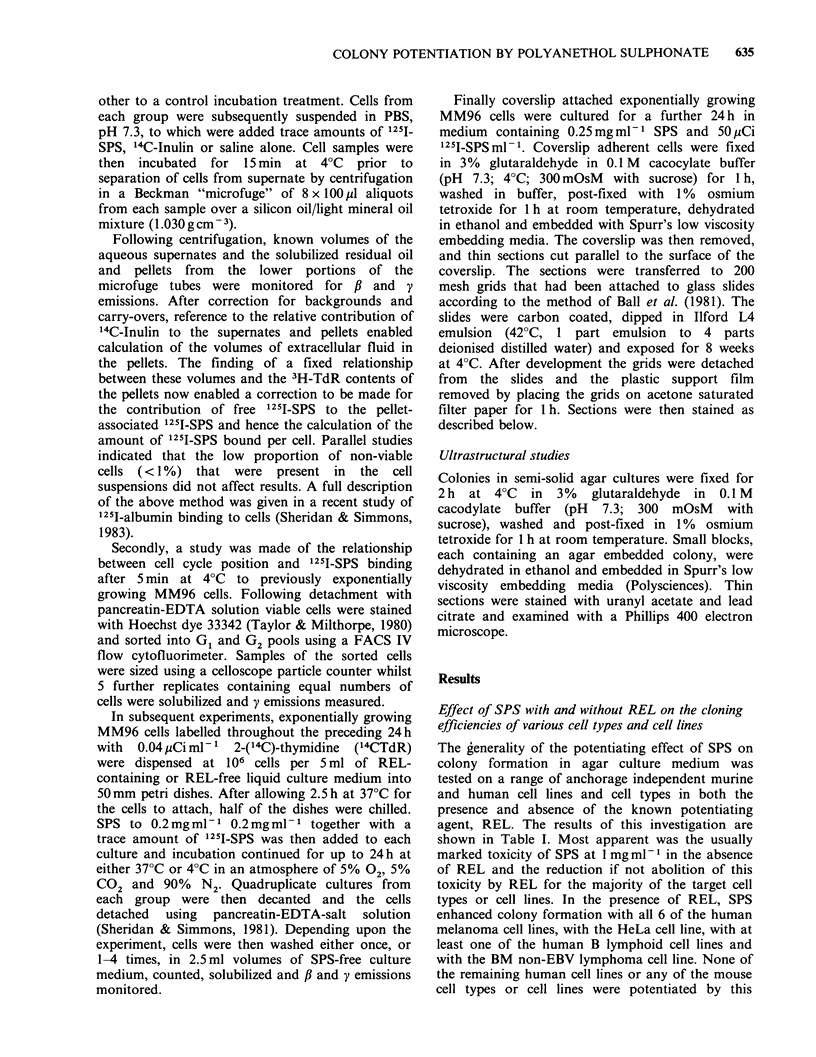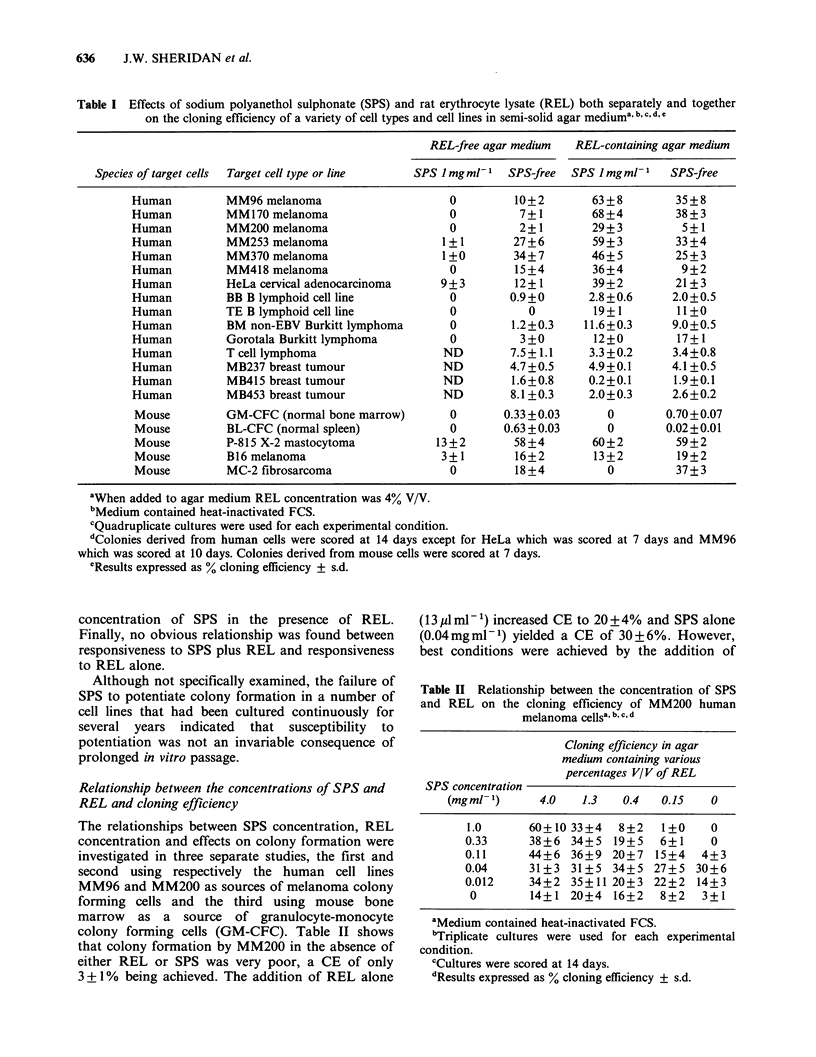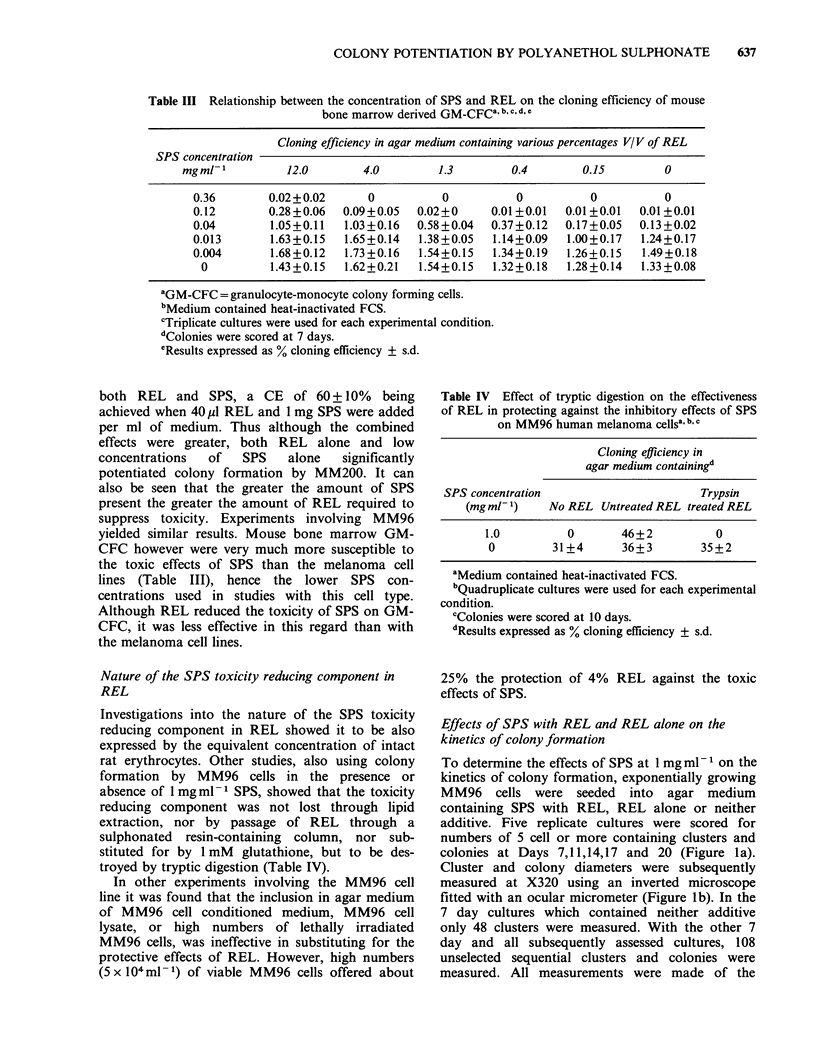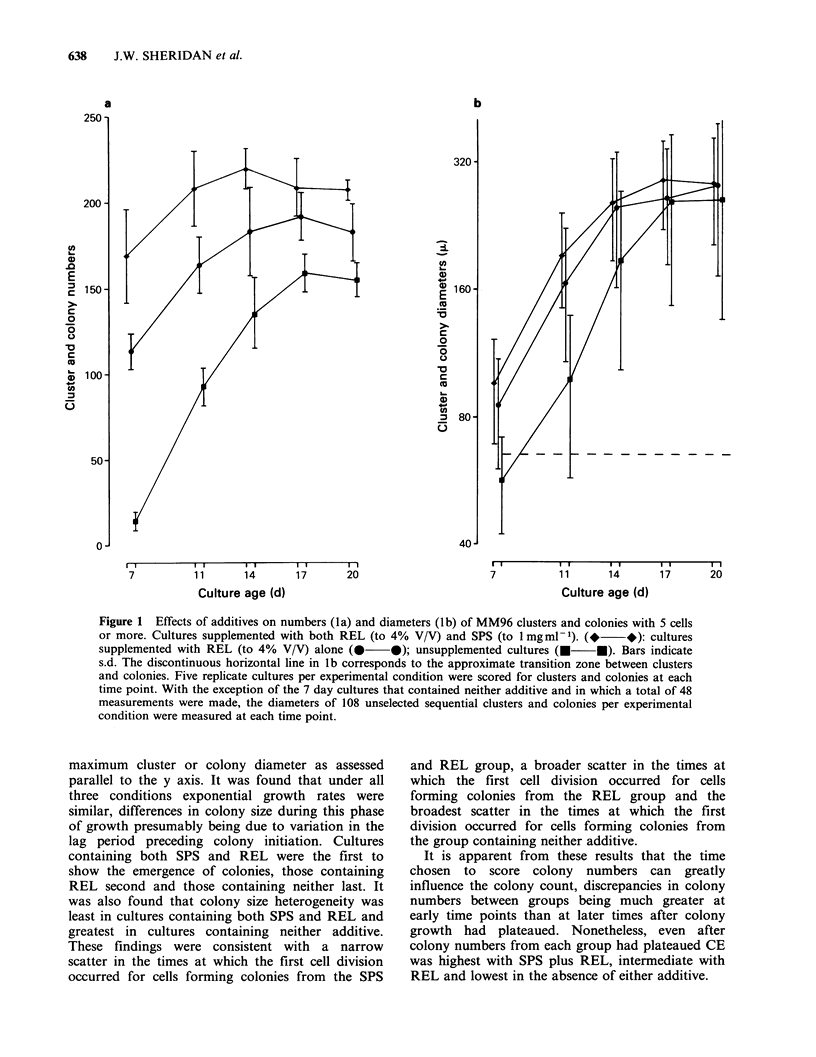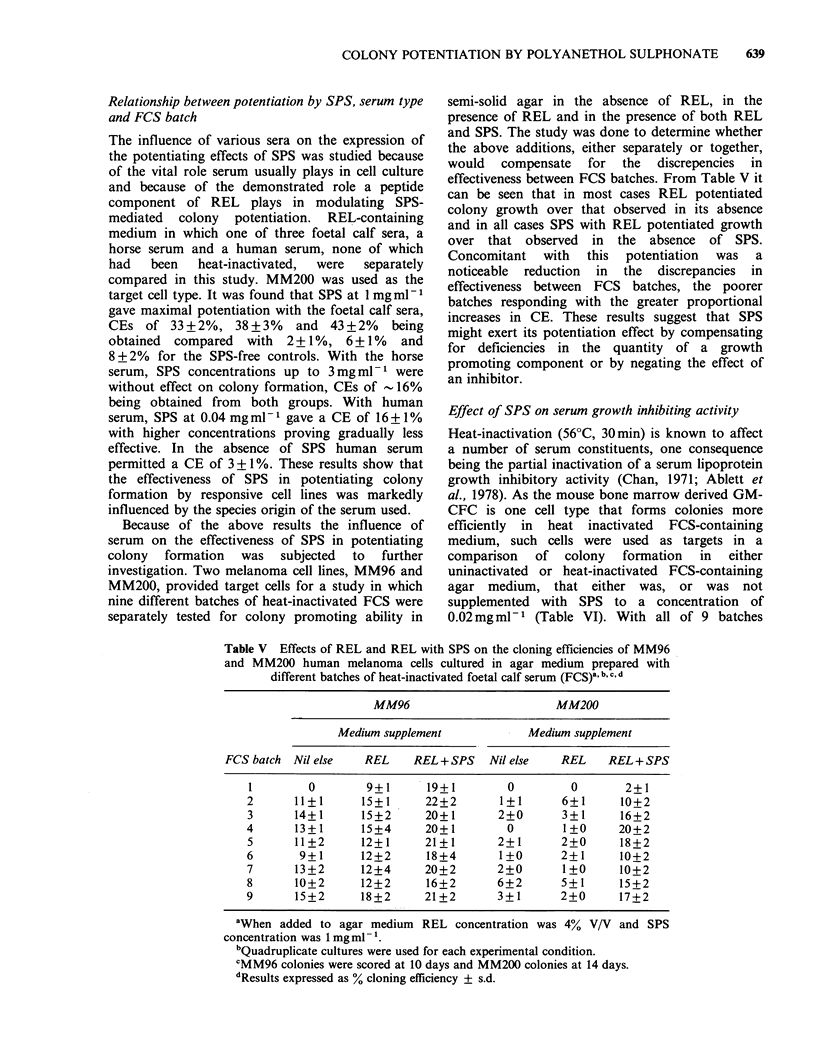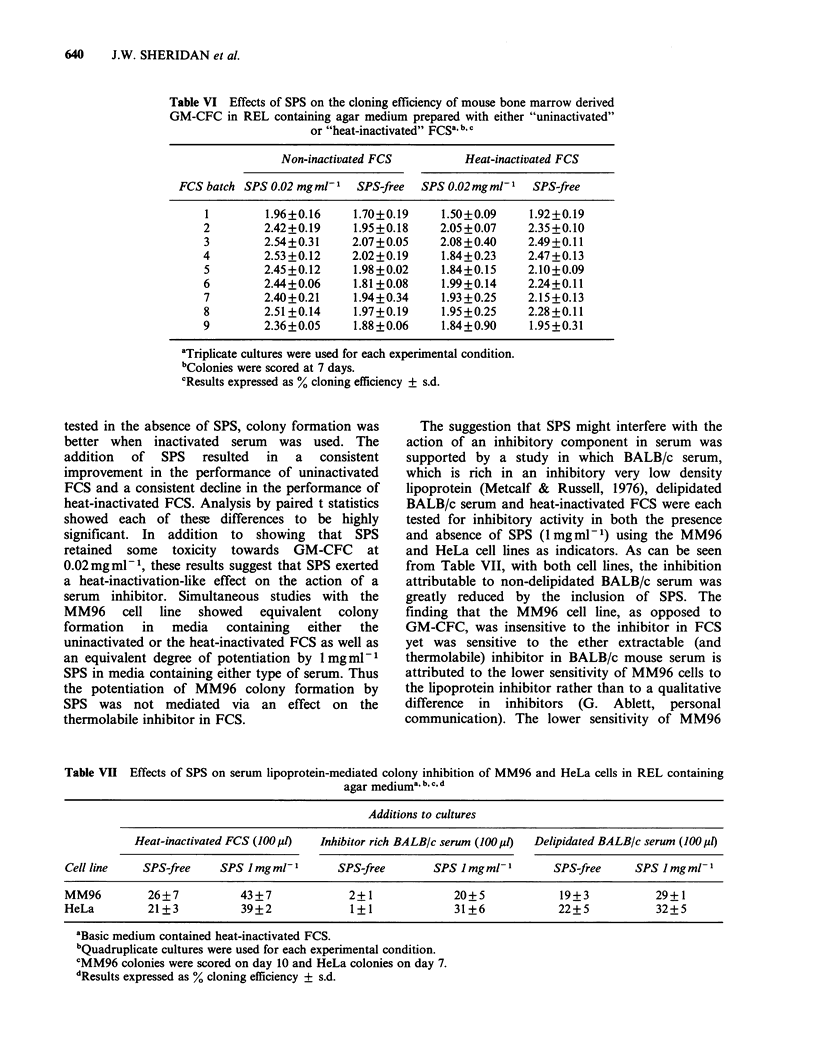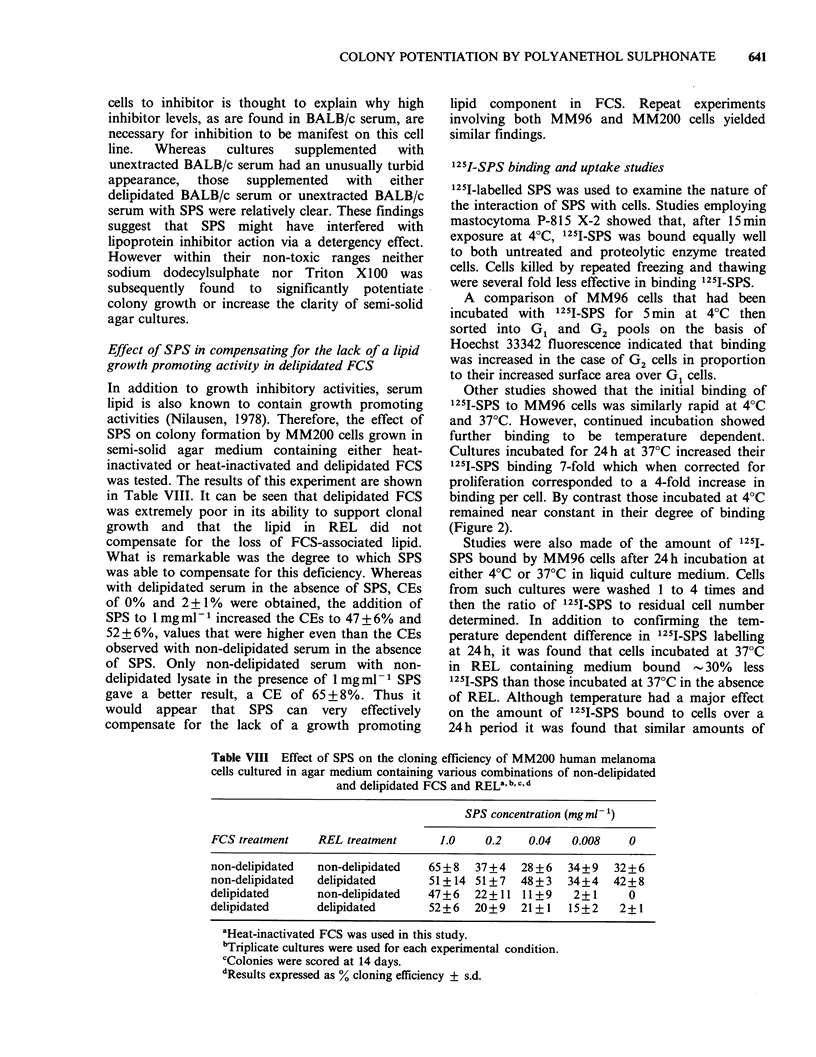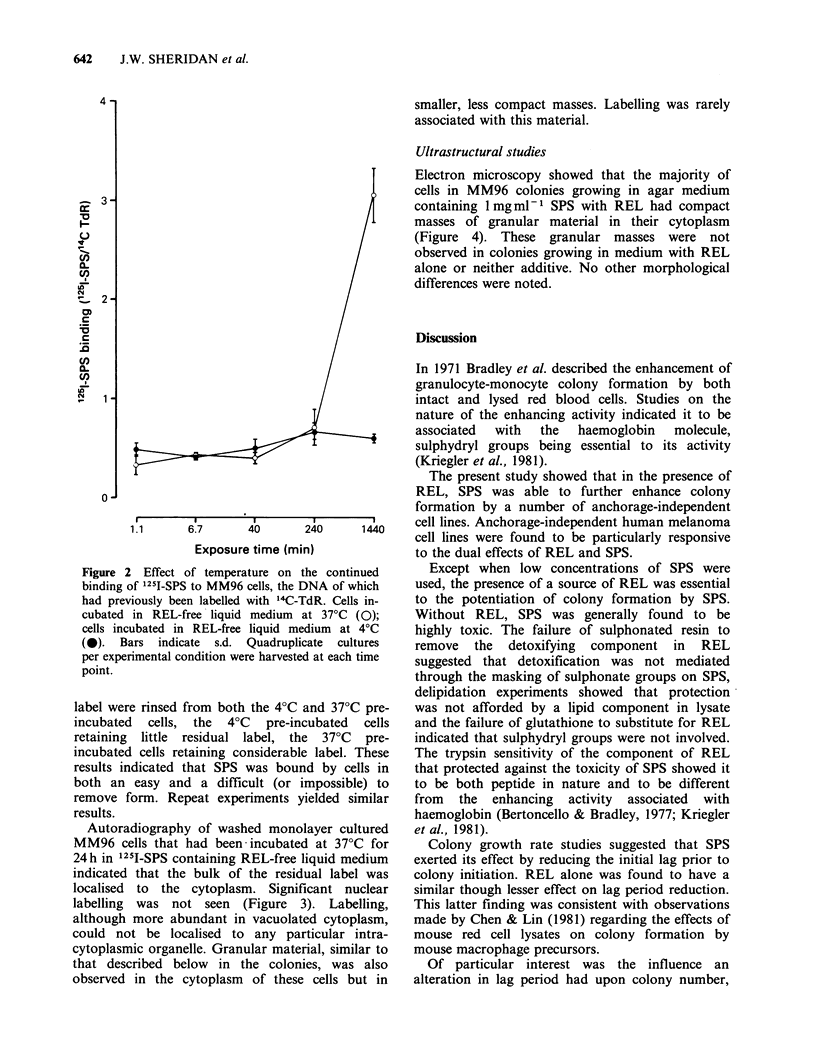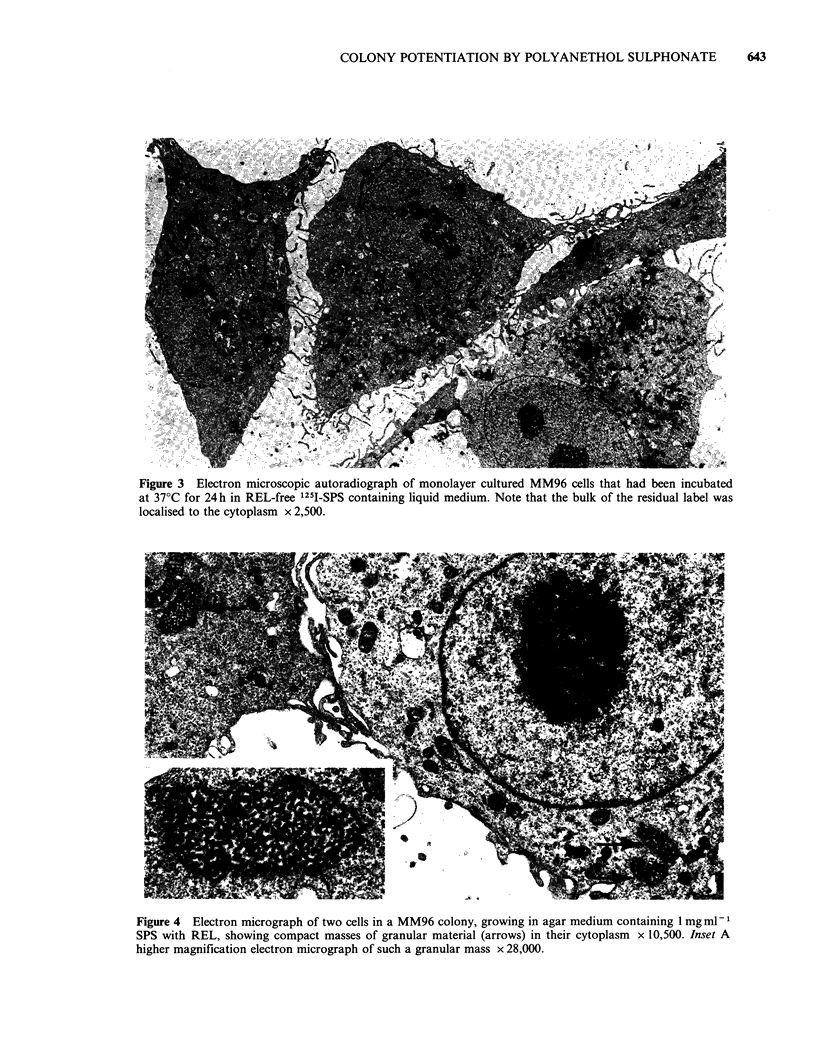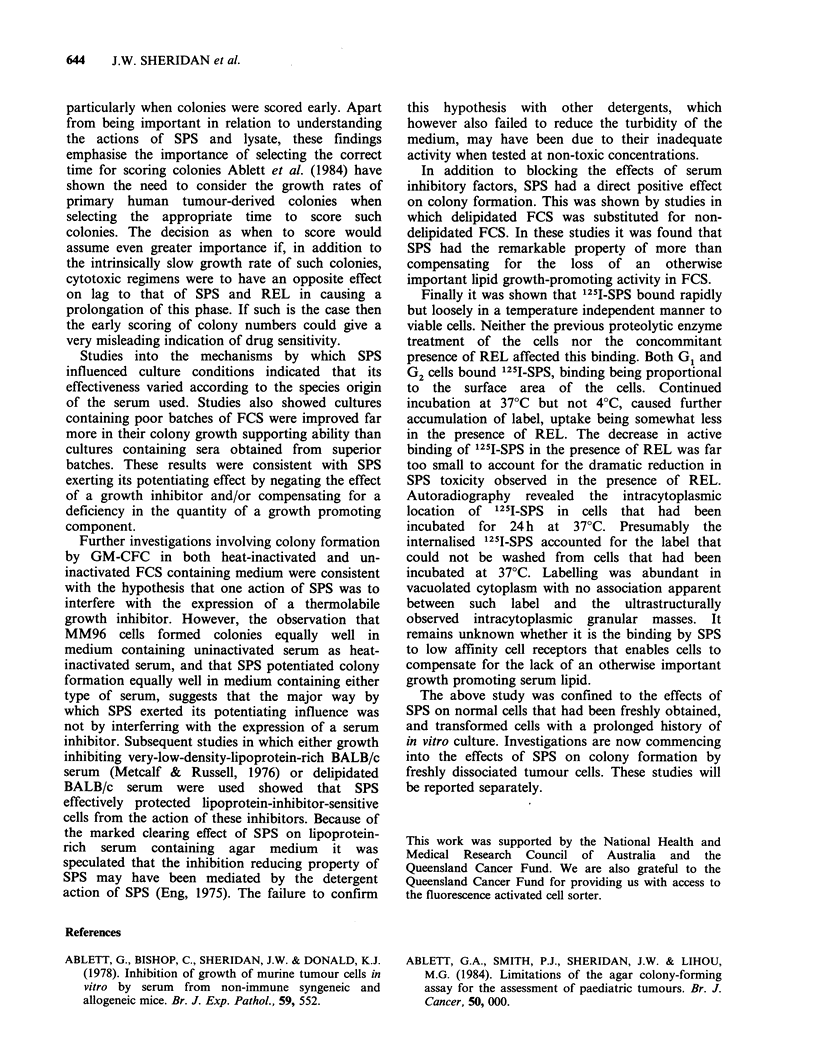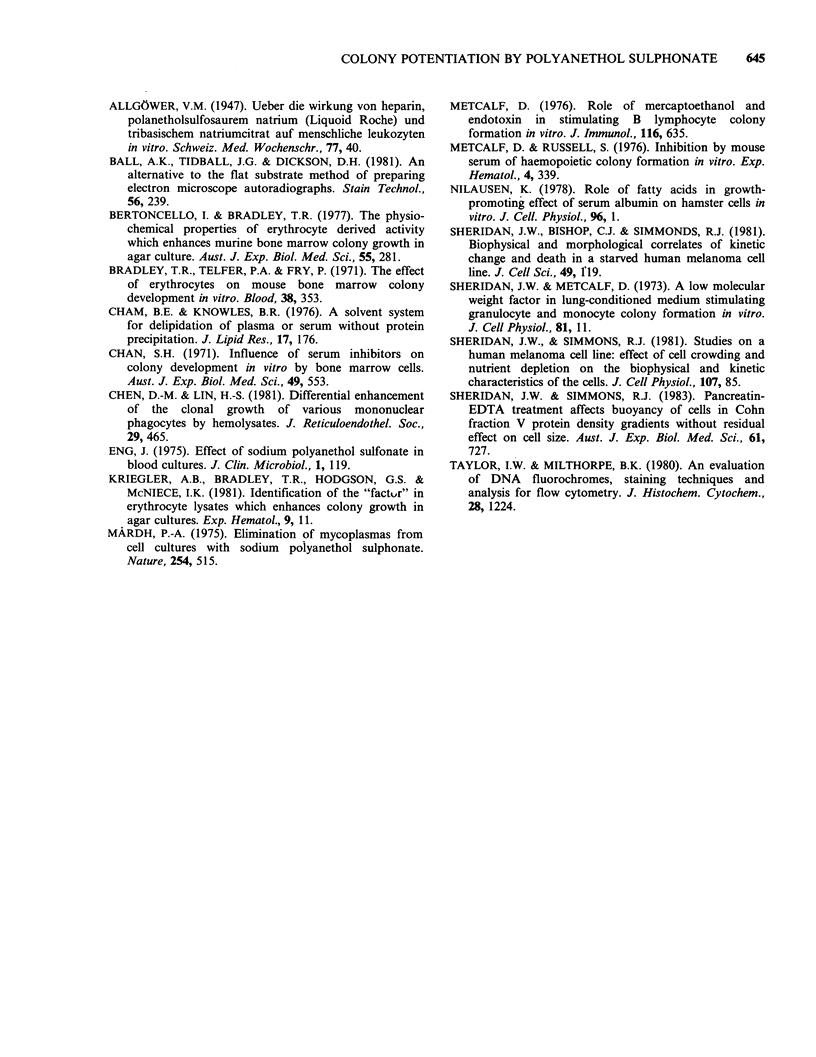# Potentiation of anchorage-independent colony formation by sodium polyanethol sulphonate.

**DOI:** 10.1038/bjc.1984.230

**Published:** 1984-11

**Authors:** J. W. Sheridan, C. J. Bishop, R. J. Simmons, C. J. Ward, K. C. Baumann

## Abstract

**Images:**


*Br. J. Cancer* (1984), **50**, 633–645

### Potentiation of anchorage-independent colony formation by sodium polyanethol sulphonate

J.W. Sheridan, C.J. Bishop, R.J. Simmons, C.J. Ward & K.C. Baumann

*Queensland Institute of Medical Research, Bramston Terrace, Herston (Brisbane) 4006, Australia.*

**Summary** Sodium polyanethol sulphonate (SPS) when incorporated into rat erythrocyte lysate (REL) containing semi-solid agar medium at $1\,mg\,ml^{-1}$. markedly enhanced colony formation by a number of anchorage-independent cell lines. REL usually needed to be included for the expression of SPS induced potentiation as in its absence SPS was generally cytotoxic. Studies suggested that SPS reduced the lag prior to colony initiation resulting in the earlier appearance of colonies and in a higher cloning efficiency. The effectiveness of SPS in potentiating colony formation by responsive cell lines was markedly influenced by the species of serum and to a lesser extent by differences between individual batches. Enhancement by SPS was greater with poorer foetal calf serum (FCS) batches than with better. This effect may have been partly due to SPS interfering with the action of a growth inhibiting serum component, possibly a lipoprotein. Studies in which delipidated FCS was substituted for normal FCS suggested that SPS was also able to compensate for the lack of a growth-promoting lipid component. Binding studies showed that initially $^{125}$I-SPS bound equally well at 4°C and 37°C with continued labelling occurring only at 37°C. Autoradiography of cells labelled at 37°C for 24 h revealed the presence of intracytoplasmic $^{125}$I-SPS.

Several polyanionic compounds were tested for effectiveness in preventing cell clumping in semi-solid agar culture medium. One of these, sodium polyanethol sulphonate (SPS), although unsatisfactory in the above regard was unexpectedly found to markedly enhance the cloning efficiency and increase the rate of appearance of MM96 human melanoma colonies grown in rat erythrocyte lysate (REL) containing agar culture medium. In addition to its effect on colony formation, SPS greatly increased the clarity of the usually somewhat turbid agar culture medium.

Polyanethol sulphonic acid is a polydisperse, though predominantly high molecular weight, polymer of *p*-methoxystyrene. As the sodium salt (SPS) it is a surface active agent that has found use as a synthetic polyanionic anticoagulant. SPS also possesses anticomplement activity and consequently has been used to inhibit serum bacteriocidal activity (Eng, 1975). It has also proved valuable in inhibiting phagocytosis (Allgöwer, 1947), and in eliminating mycoplasmal growth from cell cultures (Mårdh, 1975).

The generality of the potentiating effect of SPS on colony formation in agar culture medium was subsequently tested on a range of anchorage-independent murine and human cell lines and cell types in both the presence and absence of the known potentiating agent, REL (Bradley *et al.*, 1971; Bertoncello & Bradley, 1977; Kriegler *et al.*, 1981).

Correspondence: J.W. Sheridan
Received 9 April 1984; accepted 2 August 1984.

The present investigation into the action of SPS was made because of the possible practical value of this compound, or others like it, in promoting *in vitro* colony growth by human tumour cells, an area of potential importance to the development of predictive assays of tumour cell drug sensitivity yet currently hampered with difficulties. It was also made because of the possibility that the study would contribute to knowledge on growth regulation.

### Materials and methods

### *Cell lines and cell types*

Six human melanoma cell lines (MM96, MM170, MM200, MM253, MM370 and MM418), the HeLa cervical adenocarcinoma cell line, 3 human breast tumour cell lines (MB237, MB415 and MB453), 3 human lymphoma cell lines (the BM non-EBV Burkitt lymphoma, the Gorotala Burkitt lymphoma and an unnamed T cell lymphoma) and 2 EBV transformed B lymphoid cell lines (BB and TE) were used in this study. In addition the mouse mastocytoma cell line P-815 X-2, the mouse melanoma cell line B16, the mouse fibrosarcoma cell line MC-2 as well as cells of 2 normal lineages (bone marrow derived granulocyte-monocyte colony forming cells (GM-CFC) and spleen derived B lymphoid colony forming cells (BL-CFC)) were used. The human cell lines, each of which had been passaged in culture for a cumulative period of at least 2 years, were maintained in a modified RPMI 1640 liquid tissue culture medium (Sheridan &

© The Macmillan Press Ltd., 1984

Simmons, 1981). The mouse cell lines, each of which had been cultured for ~6 months, were grown in a similar though more concentrated medium that was prepared isoosmotic to mouse serum rather than human serum. To both media, heat-inactivated FCS (56°C, 30 min) was added to 10%. In all cases, incubation was in a fully humidified atmosphere of 5% $O_2$, 5% $CO_2$ and 90% $N_2$.

*Cell collection and cell counts*

Tumour cell lines were harvested and total and viable cell counts made according to previously described methods (Sheridan & Simmons, 1981, 1983). Mouse bone marrow and spleen cells were collected (Sheridan & Metcalf, 1973; Metcalf, 1976) immediately prior to counting and culture in semi-solid agar medium.

*Clonogenic cell assay*

The basic rat erythrocyte lysate (REL) containing agar medium has been fully described previously (Sheridan & Simmons, 1981). With the exception that on some occasions water was substituted for REL, the medium used in these studies generally conformed to that previously described. Unless stated to the contrary the medium contained heat-inactivated FCS. In studies involving mouse bone marrow or spleen cells either a source of granulocyte-monocyte colony stimulating factor (GM-CSF) (Sheridan & Metcalf, 1973) or 2-mercaptoethanol and endotoxin were included (Metcalf, 1976).

REL was prepared using a technique similar to that described by Bertoncello & Bradley (1977). Heparinised blood from August rats was centrifuged and washed three times in saline at 4°C to remove buffy coat and serum proteins. Packed erythrocytes were then mixed with 3 times their volume of chilled 0.1% acetic acid till lysis was complete. The lysate was then centrifuged 20,000 *g*, 2 h 4°C and the supernatant REL solution sterilized by membrane filtration.

Soft (0.28%) agar medium at 37°C was mixed thoroughly with the various cell suspensions to give final cell concentrations of 250 viable cells $ml^{-1}$. Exceptions were mouse bone marrow, 10,000 cells $ml^{-1}$; mouse spleen cells. 20,000 cells $ml^{-1}$; the human lymphoma cell lines, 2,500 cells $ml^{-1}$ and the human breast tumour cell lines, 10,000 cells $ml^{-1}$. One millilitre aliquots of this suspension were plated into 3 or more replicate 35 mm Petrie dishes, allowed to gel, then incubated at 37°C in a fully humidified atmosphere of 5% $O_2$, 5% $CO_2$ and 90% $N_2$ until scored. Colony counts, and on some occasions cluster counts, were made with a dissecting microscope, Aggregates of 5–50 cells were scored as clusters, those of >50 cells as colonies. With the exception of colonies derived from HeLa cells that weren scored at 7 days and colonies from MM96 cells that were scored at 10 days (unless stated otherwise), colonies derived from human cells were scored at 14 days. Colonies derived from mouse cells were scored at 7 days. Results were expressed in terms of % cloning efficiency (CE)±s.d. CE was calculated according to the formula CE = number of colonies × 100/number of cells plated.

*Rar erythrocyte lysate related experiments*

Agar medium containing intact erythrocytes was compared with medium containing the equivalent concentration of REL and medium containing neither additive. This was done to determine whether the SPS toxicity reducing component in REL was also expressed by intact erythrocytes. Because of the turbidity of cultures that incorporated erythrocytes it was necessary to induce lysis with 1 ml of 3% acetic acid per culture immediately prior to scoring for colonies.

Two ml of REL that had been passaged through a 5 ml "Amberlite" sulphonated resin column was compared with unpassaged REL for effectiveness in both potentiating colony formation in the absence of SPS and in offering protection against the toxicity of SPS.

Trypsin digested REL prepared according to the method described by Bertoncello & Bradley (1977) was tested to determine whether the SPS toxicity reducing component in REL was trypsin sensitive. Control experiments showed trypsin digested REL to be non-toxic. MM96 cells were used as targets in all 3 of the above experiments.

*Delipidation of REL and FCS*

FCS and REL were delipidated according to the method described by Cham & Knowles (1976).

*$^{125}$I-SPS cell binding studies*

SPS was labelled with $^{125}$I by means of 1,3,4,6-tetrachloro-3α, 6α diphenylglycoluril (Iodogen, Pierce) using the method described by the manufacturer.

Firstly, a comparison was made of the degree of binding of $^{125}$I-SPS after 15 min at 4°C to both previously untreated and pancreatin-EDTA treated viable or subsequently freeze-thaw killed mastocytoma cells. Exponentially growing cells, labelled throughout the 24 h period prior to harvest with 0.2 μCi $ml^{-1}$ (methyl-$^3$H)-thymidine ($^3$H-TdR) were divided into two groups, one of which was exposed to pancreatin-EDTA treatment and the other to a control incubation treatment. Cells from each group were subsequently suspended in PBS, pH 7.3, to which were added trace amounts of $^{125}$I-SPS, $^{14}$C-Inulin or saline alone. Cell samples were then incubated for 15 min at 4°C prior to separation of cells from supernate by centrifugation in a Beckman "microfuge" of $8 \times 100\,\mu l$ aliquots from each sample over a silicon oil/light mineral oil mixture (1.030 g cm$^{-3}$).

Following centrifugation, known volumes of the aqueous supernates and the solubilized residual oil and pellets from the lower portions of the microfuge tubes were monitored for $\beta$ and $\gamma$ emissions. After correction for backgrounds and carry-overs, reference to the relative contribution of $^{14}$C-Inulin to the supernates and pellets enabled calculation of the volumes of extracellular fluid in the pellets. The finding of a fixed relationship between these volumes and the $^{3}$H-TdR contents of the pellets now enabled a correction to be made for the contribution of free $^{125}$I-SPS to the pellet-associated $^{125}$I-SPS and hence the calculation of the amount of $^{125}$I-SPS bound per cell. Parallel studies indicated that the low proportion of non-viable cells (<1%) that were present in the cell suspensions did not affect results. A full description of the above method was given in a recent study of $^{125}$I-albumin binding to cells (Sheridan & Simmons, 1983).

Secondly, a study was made of the relationship between cell cycle position and $^{125}$I-SPS binding after 5 min at 4°C to previously exponentially growing MM96 cells. Following detachment with pancreatin-EDTA solution viable cells were stained with Hoechst dye 33342 (Taylor & Milthorpe, 1980) and sorted into $G_1$ and $G_2$ pools using a FACS IV flow cytofluorimeter. Samples of the sorted cells were sized using a celloscope particle counter whilst 5 further replicates containing equal numbers of cells were solubilized and $\gamma$ emissions measured.

In subsequent experiments, exponentially growing MM96 cells labelled throughout the preceding 24 h with $0.04\,\mu$Ci ml$^{-1}$ 2-($^{14}$C)-thymidine ($^{14}$CTdR) were dispensed at $10^6$ cells per 5 ml of REL-containing or REL-free liquid culture medium into 50 mm petri dishes. After allowing 2.5 h at 37°C for the cells to attach, half of the dishes were chilled. SPS to 0.2 mg ml$^{-1}$ 0.2 mg ml$^{-1}$ together with a trace amount of $^{125}$I-SPS was then added to each culture and incubation continued for up to 24 h at either 37°C or 4°C in an atmosphere of 5% $O_2$, 5% $CO_2$ and 90% $N_2$. Quadruplicate cultures from each group were then decanted and the cells detached using pancreatin-EDTA-salt solution (Sheridan & Simmons, 1981). Depending upon the experiment, cells were then washed either once, or 1–4 times, in 2.5 ml volumes of SPS-free culture medium, counted, solubilized and $\beta$ and $\gamma$ emissions monitored.

Finally coverslip attached exponentially growing MM96 cells were cultured for a further 24 h in medium containing 0.25 mg ml$^{-1}$ SPS and $50\,\mu$Ci $^{125}$I-SPS ml$^{-1}$. Coverslip adherent cells were fixed in 3% glutaraldehyde in 0.1 M cacocylate buffer (pH 7.3; 4°C; 300 mOsM with sucrose) for 1 h, washed in buffer, post-fixed with 1% osmium tetroxide for 1 h at room temperature, dehydrated in ethanol and embedded with Spurr's low viscosity embedding media. The coverslip was then removed, and thin sections cut parallel to the surface of the coverslip. The sections were transferred to 200 mesh grids that had been attached to glass slides according to the method of Ball *et al.* (1981). The slides were carbon coated, dipped in Ilford L4 emulsion (42°C, 1 part emulsion to 4 parts deionised distilled water) and exposed for 8 weeks at 4°C. After development the grids were detached from the slides and the plastic support film removed by placing the grids on acetone saturated filter paper for 1 h. Sections were then stained as described below.

*Ultrastructural studies*

Colonies in semi-solid agar cultures were fixed for 2 h at 4°C in 3% glutaraldehyde in 0.1 M cacodylate buffer (pH 7.3; 300 mOsM with sucrose), washed and post-fixed in 1% osmium tetroxide for 1 h at room temperature. Small blocks, each containing an agar embedded colony, were dehydrated in ethanol and embedded in Spurr's low viscosity embedding media (Polysciences). Thin sections were stained with uranyl acetate and lead citrate and examined with a Phillips 400 electron microscope.

### Results

*Effect of SPS with and without REL on the cloning efficiencies of various cell types and cell lines*

The generality of the potentiating effect of SPS on colony formation in agar culture medium was tested on a range of anchorage independent murine and human cell lines and cell types in both the presence and absence of the known potentiating agent, REL. The results of this investigation are shown in Table I. Most apparent was the usually marked toxicity of SPS at 1 mg ml$^{-1}$ in the absence of REL and the reduction if not abolition of this toxicity by REL for the majority of the target cell types or cell lines. In the presence of REL, SPS enhanced colony formation with all 6 of the human melanoma cell lines, with the HeLa cell line, with at least one of the human B lymphoid cell lines and with the BM non-EBV lymphoma cell line. None of the remaining human cell lines or any of the mouse cell types or cell lines were potentiated by this

**Table I** Effects of sodium polyanethol sulphonate (SPS) and rat erythrocyte lysate (REL) both separately and together on the cloning efficiency of a variety of cell types and cell lines in semi-solid agar medium[a,b,c,d,e]

| Species of target cells | Target cell type or line | *REL-free agar medium* SPS 1 mg ml$^{-1}$ | *REL-free agar medium* SPS-free | *REL-containing agar medium* SPS 1 mg ml$^{-1}$ | *REL-containing agar medium* SPS-free |
|---|---|---|---|---|---|
| Human | MM96 melanoma | 0 | 10±2 | 63±8 | 35±8 |
| Human | MM170 melanoma | 0 | 7±1 | 68±4 | 38±3 |
| Human | MM200 melanoma | 0 | 2±1 | 29±3 | 5±1 |
| Human | MM253 melanoma | 1±1 | 27±6 | 59±3 | 33±4 |
| Human | MM370 melanoma | 1±0 | 34±7 | 46±5 | 25±3 |
| Human | MM418 melanoma | 0 | 15±4 | 36±4 | 9±2 |
| Human | HeLa cervical adenocarcinoma | 9±3 | 12±1 | 39±2 | 21±3 |
| Human | BB B lymphoid cell line | 0 | 0.9±0 | 2.8±0.6 | 2.0±0.5 |
| Human | TE B lymphoid cell line | 0 | 0 | 19±1 | 11±0 |
| Human | BM non-EBV Burkitt lymphoma | 0 | 1.2±0.3 | 11.6±0.3 | 9.0±0.5 |
| Human | Gorotala Burkitt lymphoma | 0 | 3±0 | 12±0 | 17±1 |
| Human | T cell lymphoma | ND | 7.5±1.1 | 3.3±0.2 | 3.4±0.8 |
| Human | MB237 breast tumour | ND | 4.7±0.5 | 4.9±0.1 | 4.1±0.5 |
| Human | MB415 breast tumour | ND | 1.6±0.8 | 0.2±0.1 | 1.9±0.1 |
| Human | MB453 breast tumour | ND | 8.1±0.3 | 2.0±0.3 | 2.6±0.2 |
| Mouse | GM-CFC (normal bone marrow) | 0 | 0.33±0.03 | 0 | 0.70±0.07 |
| Mouse | BL-CFC (normal spleen) | 0 | 0.63±0.03 | 0 | 0.02±0.01 |
| Mouse | P-815 X-2 mastocytoma | 13±2 | 58±4 | 60±2 | 59±2 |
| Mouse | B16 melanoma | 3±1 | 16±2 | 13±2 | 19±2 |
| Mouse | MC-2 fibrosarcoma | 0 | 18±4 | 0 | 37±3 |

[a]When added to agar medium REL concentration was 4% V/V.
[b]Medium contained heat-inactivated FCS.
[c]Quadruplicate cultures were used for each experimental condition.
[d]Colonies derived from human cells were scored at 14 days except for HeLa which was scored at 7 days and MM96 which was scored at 10 days. Colonies derived from mouse cells were scored at 7 days.
[e]Results expressed as % cloning efficiency ± s.d.

concentration of SPS in the presence of REL. Finally, no obvious relationship was found between responsiveness to SPS plus REL and responsiveness to REL alone.

Although not specifically examined, the failure of SPS to potentiate colony formation in a number of cell lines that had been cultured continuously for several years indicated that susceptibility to potentiation was not an invariable consequence of prolonged *in vitro* passage.

*Relationship between the concentrations of SPS and REL and cloning efficiency*

The relationships between SPS concentration, REL concentration and effects on colony formation were investigated in three separate studies, the first and second using respectively the human cell lines MM96 and MM200 as sources of melanoma colony forming cells and the third using mouse bone marrow as a source of granulocyte-monocyte colony forming cells (GM-CFC). Table II shows that colony formation by MM200 in the absence of either REL or SPS was very poor, a CE of only 3±1% being achieved. The addition of REL alone (13 μl ml$^{-1}$) increased CE to 20±4% and SPS alone (0.04 mg ml$^{-1}$) yielded a CE of 30±6%. However, best conditions were achieved by the addition of

**Table II** Relationship between the concentration of SPS and REL on the cloning efficiency of MM200 human melanoma cells[a,b,c,d]

| SPS concentration (mg ml$^{-1}$) | *Cloning efficiency in agar medium containing various percentages V/V of REL* 4.0 | 1.3 | 0.4 | 0.15 | 0 |
|---|---|---|---|---|---|
| 1.0 | 60±10 | 33±4 | 8±2 | 1±0 | 0 |
| 0.33 | 38±6 | 34±5 | 19±5 | 6±1 | 0 |
| 0.11 | 44±6 | 36±9 | 20±7 | 15±4 | 4±3 |
| 0.04 | 31±3 | 31±5 | 34±5 | 27±5 | 30±6 |
| 0.012 | 34±2 | 35±11 | 20±3 | 22±2 | 14±3 |
| 0 | 14±1 | 20±4 | 16±2 | 8±2 | 3±1 |

[a]Medium contained heat-inactivated FCS.
[b]Triplicate cultures were used for each experimental condition.
[c]Cultures were scored at 14 days.
[d]Results expressed as % cloning efficiency ± s.d.

**Table III** Relationship between the concentration of SPS and REL on the cloning efficiency of mouse bone marrow derived GM-CFC[a,b,c,d,e]

| *SPS concentration* | *Cloning efficiency in agar medium containing various percentages V/V of REL* | | | | | |
|---|---|---|---|---|---|---|
| *mg ml⁻¹* | *12.0* | *4.0* | *1.3* | *0.4* | *0.15* | *0* |
| 0.36 | 0.02±0.02 | 0 | 0 | 0 | 0 | 0 |
| 0.12 | 0.28±0.06 | 0.09±0.05 | 0.02±0 | 0.01±0.01 | 0.01±0.01 | 0.01±0.01 |
| 0.04 | 1.05±0.11 | 1.03±0.16 | 0.58±0.04 | 0.37±0.12 | 0.17±0.05 | 0.13±0.02 |
| 0.013 | 1.63±0.15 | 1.65±0.14 | 1.38±0.05 | 1.14±0.09 | 1.00±0.17 | 1.24±0.17 |
| 0.004 | 1.68±0.12 | 1.73±0.16 | 1.54±0.15 | 1.34±0.19 | 1.26±0.15 | 1.49±0.18 |
| 0 | 1.43±0.15 | 1.62±0.21 | 1.54±0.15 | 1.32±0.18 | 1.28±0.14 | 1.33±0.08 |

[a]GM-CFC = granulocyte-monocyte colony forming cells.
[b]Medium contained heat-inactivated FCS.
[c]Triplicate cultures were used for each experimental condition.
[d]Colonies were scored at 7 days.
[e]Results expressed as % cloning efficiency ± s.d.

both REL and SPS, a CE of 60±10% being achieved when 40 μl REL and 1 mg SPS were added per ml of medium. Thus although the combined effects were greater, both REL alone and low concentrations of SPS alone significantly potentiated colony formation by MM200. It can also be seen that the greater the amount of SPS present the greater the amount of REL required to suppress toxicity. Experiments involving MM96 yielded similar results. Mouse bone marrow GM-CFC however were very much more susceptible to the toxic effects of SPS than the melanoma cell lines (Table III), hence the lower SPS concentrations used in studies with this cell type. Although REL reduced the toxicity of SPS on GM-CFC, it was less effective in this regard than with the melanoma cell lines.

*Nature of the SPS toxicity reducing component in REL*

Investigations into the nature of the SPS toxicity reducing component in REL showed it to be also expressed by the equivalent concentration of intact rat erythrocytes. Other studies, also using colony formation by MM96 cells in the presence or absence of 1 mg ml⁻¹ SPS, showed that the toxicity reducing component was not lost through lipid extraction, nor by passage of REL through a sulphonated resin-containing column, nor substituted for by 1 mM glutathione, but to be destroyed by tryptic digestion (Table IV).

In other experiments involving the MM96 cell line it was found that the inclusion in agar medium of MM96 cell conditioned medium, MM96 cell lysate, or high numbers of lethally irradiated MM96 cells, was ineffective in substituting for the protective effects of REL. However, high numbers ($5 \times 10^4$ ml⁻¹) of viable MM96 cells offered about

**Table IV** Effect of tryptic digestion on the effectiveness of REL in protecting against the inhibitory effects of SPS on MM96 human melanoma cells[a,b,c]

| *SPS concentration (mg ml⁻¹)* | *Cloning efficiency in agar medium containing[d]* No REL | Untreated REL | Trypsin treated REL |
|---|---|---|---|
| 1.0 | 0 | 46±2 | 0 |
| 0 | 31±4 | 36±3 | 35±2 |

[a]Medium contained heat-inactivated FCS.
[b]Quadruplicate cultures were used for each experimental condition.
[c]Colonies were scored at 10 days.
[d]Results expressed as % cloning efficiency ± s.d.

25% the protection of 4% REL against the toxic effects of SPS.

*Effects of SPS with REL and REL alone on the kinetics of colony formation*

To determine the effects of SPS at 1 mg ml⁻¹ on the kinetics of colony formation, exponentially growing MM96 cells were seeded into agar medium containing SPS with REL, REL alone or neither additive. Five replicate cultures were scored for numbers of 5 cell or more containing clusters and colonies at Days 7,11,14,17 and 20 (Figure 1a). Cluster and colony diameters were subsequently measured at X320 using an inverted microscope fitted with an ocular micrometer (Figure 1b). In the 7 day cultures which contained neither additive only 48 clusters were measured. With the other 7 day and all subsequently assessed cultures, 108 unselected sequential clusters and colonies were measured. All measurements were made of the

**Figure 1** Effects of additives on numbers (1a) and diameters (1b) of MM96 clusters and colonies with 5 cells or more. Cultures supplemented with both REL (to 4% V/V) and SPS (to 1 mg ml$^{-1}$). (◆——◆): cultures supplemented with REL (to 4% V/V) alone (●——●); unsupplemented cultures (■——■). Bars indicate s.d. The discontinuous horizontal line in 1b corresponds to the approximate transition zone between clusters and colonies. Five replicate cultures per experimental condition were scored for clusters and colonies at each time point. With the exception of the 7 day cultures that contained neither additive and in which a total of 48 measurements were made, the diameters of 108 unselected sequential clusters and colonies per experimental condition were measured at each time point.

maximum cluster or colony diameter as assessed parallel to the y axis. It was found that under all three conditions exponential growth rates were similar, differences in colony size during this phase of growth presumably being due to variation in the lag period preceding colony initiation. Cultures containing both SPS and REL were the first to show the emergence of colonies, those containing REL second and those containing neither last. It was also found that colony size heterogeneity was least in cultures containing both SPS and REL and greatest in cultures containing neither additive. These findings were consistent with a narrow scatter in the times at which the first cell division occurred for cells forming colonies from the SPS and REL group, a broader scatter in the times at which the first cell division occurred for cells forming colonies from the REL group and the broadest scatter in the times at which the first division occurred for cells forming colonies from the group containing neither additive.

It is apparent from these results that the time chosen to score colony numbers can greatly influence the colony count, discrepancies in colony numbers between groups being much greater at early time points than at later times after colony growth had plateaued. Nonetheless, even after colony numbers from each group had plateaued CE was highest with SPS plus REL, intermediate with REL and lowest in the absence of either additive.

*Relationship between potentiation by SPS, serum type and FCS batch*

The influence of various sera on the expression of the potentiating effects of SPS was studied because of the vital role serum usually plays in cell culture and because of the demonstrated role a peptide component of REL plays in modulating SPS-mediated colony potentiation. REL-containing medium in which one of three foetal calf sera, a horse serum and a human serum, none of which had been heat-inactivated, were separately compared in this study. MM200 was used as the target cell type. It was found that SPS at $1\,mg\,ml^{-1}$ gave maximal potentiation with the foetal calf sera, CEs of $33\pm2\%$, $38\pm3\%$ and $43\pm2\%$ being obtained compared with $2\pm1\%$, $6\pm1\%$ and $8\pm2\%$ for the SPS-free controls. With the horse serum, SPS concentrations up to $3\,mg\,ml^{-1}$ were without effect on colony formation, CEs of $\sim16\%$ being obtained from both groups. With human serum, SPS at $0.04\,mg\,ml^{-1}$ gave a CE of $16\pm1\%$ with higher concentrations proving gradually less effective. In the absence of SPS human serum permitted a CE of $3\pm1\%$. These results show that the effectiveness of SPS in potentiating colony formation by responsive cell lines was markedly influenced by the species origin of the serum used.

Because of the above results the influence of serum on the effectiveness of SPS in potentiating colony formation was subjected to further investigation. Two melanoma cell lines, MM96 and MM200, provided target cells for a study in which nine different batches of heat-inactivated FCS were separately tested for colony promoting ability in semi-solid agar in the absence of REL, in the presence of REL and in the presence of both REL and SPS. The study was done to determine whether the above additions, either separately or together, would compensate for the discrepencies in effectiveness between FCS batches. From Table V it can be seen that in most cases REL potentiated colony growth over that observed in its absence and in all cases SPS with REL potentiated growth over that observed in the absence of SPS. Concomitant with this potentiation was a noticeable reduction in the discrepancies in effectiveness between FCS batches, the poorer batches responding with the greater proportional increases in CE. These results suggest that SPS might exert its potentiation effect by compensating for deficiencies in the quantity of a growth promoting component or by negating the effect of an inhibitor.

*Effect of SPS on serum growth inhibiting activity*

Heat-inactivation (56°C, 30 min) is known to affect a number of serum constituents, one consequence being the partial inactivation of a serum lipoprotein growth inhibitory activity (Chan, 1971; Ablett *et al.*, 1978). As the mouse bone marrow derived GM-CFC is one cell type that forms colonies more efficiently in heat inactivated FCS-containing medium, such cells were used as targets in a comparison of colony formation in either uninactivated or heat-inactivated FCS-containing agar medium, that either was, or was not supplemented with SPS to a concentration of $0.02\,mg\,ml^{-1}$ (Table VI). With all of 9 batches

**Table V** Effects of REL and REL with SPS on the cloning efficiencies of MM96 and MM200 human melanoma cells cultured in agar medium prepared with different batches of heat-inactivated foetal calf serum (FCS)[a,b,c,d]

| | *MM96* | | | *MM200* | | |
|---|---|---|---|---|---|---|
| | *Medium supplement* | | | *Medium supplement* | | |
| *FCS batch* | *Nil else* | *REL* | *REL+SPS* | *Nil else* | *REL* | *REL+SPS* |
| 1 | 0 | 9±1 | 19±1 | 0 | 0 | 2±1 |
| 2 | 11±1 | 15±1 | 22±2 | 1±1 | 6±1 | 10±2 |
| 3 | 14±1 | 15±2 | 20±1 | 2±0 | 3±1 | 16±2 |
| 4 | 13±1 | 15±4 | 20±1 | 0 | 1±0 | 20±2 |
| 5 | 11±2 | 12±1 | 21±1 | 2±1 | 2±0 | 18±2 |
| 6 | 9±1 | 12±2 | 18±4 | 1±0 | 2±1 | 10±2 |
| 7 | 13±2 | 12±4 | 20±2 | 2±0 | 1±0 | 10±2 |
| 8 | 10±2 | 12±2 | 16±2 | 6±2 | 5±1 | 15±2 |
| 9 | 15±2 | 18±2 | 21±2 | 3±1 | 2±0 | 17±2 |

[a]When added to agar medium REL concentration was 4% V/V and SPS concentration was $1\,mg\,ml^{-1}$.
[b]Quadruplicate cultures were used for each experimental condition.
[c]MM96 colonies were scored at 10 days and MM200 colonies at 14 days.
[d]Results expressed as % cloning efficiency ± s.d.

**Table VI** Effects of SPS on the cloning efficiency of mouse bone marrow derived GM-CFC in REL containing agar medium prepared with either "uninactivated" or "heat-inactivated" FCS[a, b, c]

| | *Non-inactivated FCS* | | *Heat-inactivated FCS* | |
|---|---|---|---|---|
| *FCS batch* | *SPS 0.02 mg ml⁻¹* | *SPS-free* | *SPS 0.02 mg ml⁻¹* | *SPS-free* |
| 1 | 1.96±0.16 | 1.70±0.19 | 1.50±0.09 | 1.92±0.19 |
| 2 | 2.42±0.19 | 1.95±0.18 | 2.05±0.07 | 2.35±0.10 |
| 3 | 2.54±0.31 | 2.07±0.05 | 2.08±0.40 | 2.49±0.11 |
| 4 | 2.53±0.12 | 2.02±0.19 | 1.84±0.23 | 2.47±0.13 |
| 5 | 2.45±0.12 | 1.98±0.02 | 1.84±0.15 | 2.10±0.09 |
| 6 | 2.44±0.06 | 1.81±0.08 | 1.99±0.14 | 2.24±0.11 |
| 7 | 2.40±0.21 | 1.94±0.34 | 1.93±0.25 | 2.15±0.13 |
| 8 | 2.51±0.14 | 1.97±0.19 | 1.95±0.25 | 2.28±0.11 |
| 9 | 2.36±0.05 | 1.88±0.06 | 1.84±0.90 | 1.95±0.31 |

[a]Triplicate cultures were used for each experimental condition.
[b]Colonies were scored at 7 days.
[c]Results expressed as % cloning efficiency ± s.d.

tested in the absence of SPS, colony formation was better when inactivated serum was used. The addition of SPS resulted in a consistent improvement in the performance of uninactivated FCS and a consistent decline in the performance of heat-inactivated FCS. Analysis by paired t statistics showed each of these differences to be highly significant. In addition to showing that SPS retained some toxicity towards GM-CFC at $0.02 \mathrm{mg\,ml^{-1}}$, these results suggest that SPS exerted a heat-inactivation-like effect on the action of a serum inhibitor. Simultaneous studies with the MM96 cell line showed equivalent colony formation in media containing either the uninactivated or the heat-inactivated FCS as well as an equivalent degree of potentiation by $1 \mathrm{mg\,ml^{-1}}$ SPS in media containing either type of serum. Thus the potentiation of MM96 colony formation by SPS was not mediated via an effect on the thermolabile inhibitor in FCS.

The suggestion that SPS might interfere with the action of an inhibitory component in serum was supported by a study in which BALB/c serum, which is rich in an inhibitory very low density lipoprotein (Metcalf & Russell, 1976), delipidated BALB/c serum and heat-inactivated FCS were each tested for inhibitory activity in both the presence and absence of SPS ($1 \mathrm{mg\,ml^{-1}}$) using the MM96 and HeLa cell lines as indicators. As can be seen from Table VII, with both cell lines, the inhibition attributable to non-delipidated BALB/c serum was greatly reduced by the inclusion of SPS. The finding that the MM96 cell line, as opposed to GM-CFC, was insensitive to the inhibitor in FCS yet was sensitive to the ether extractable (and thermolabile) inhibitor in BALB/c mouse serum is attributed to the lower sensitivity of MM96 cells to the lipoprotein inhibitor rather than to a qualitative difference in inhibitors (G. Ablett, personal communication). The lower sensitivity of MM96

**Table VII** Effects of SPS on serum lipoprotein-mediated colony inhibition of MM96 and HeLa cells in REL containing agar medium[a, b, c, d]

| | *Additions to cultures* | | | | | |
|---|---|---|---|---|---|---|
| | *Heat-inactivated FCS (100 µl)* | | *Inhibitor rich BALB/c serum (100 µl)* | | *Delipidated BALB/c serum (100 µl)* | |
| *Cell line* | *SPS-free* | *SPS 1 mg ml⁻¹* | *SPS-free* | *SPS 1 mg ml⁻¹* | *SPS-free* | *SPS 1 mg ml⁻¹* |
| MM96 | 26±7 | 43±7 | 2±1 | 20±5 | 19±3 | 29±1 |
| HeLa | 21±3 | 39±2 | 1±1 | 31±6 | 22±5 | 32±5 |

[a]Basic medium contained heat-inactivated FCS.
[b]Quadruplicate cultures were used for each experimental condition.
[c]MM96 colonies were scored on day 10 and HeLa colonies on day 7.
[d]Results expressed as % cloning efficiency ± s.d.

cells to inhibitor is thought to explain why high inhibitor levels, as are found in BALB/c serum, are necessary for inhibition to be manifest on this cell line. Whereas cultures supplemented with unextracted BALB/c serum had an unusually turbid appearance, those supplemented with either delipidated BALB/c serum or unextracted BALB/c serum with SPS were relatively clear. These findings suggest that SPS might have interfered with lipoprotein inhibitor action via a detergency effect. However within their non-toxic ranges neither sodium dodecylsulphate nor Triton X100 was subsequently found to significantly potentiate colony growth or increase the clarity of semi-solid agar cultures.

*Effect of SPS in compensating for the lack of a lipid growth promoting activity in delipidated FCS*

In addition to growth inhibitory activities, serum lipid is also known to contain growth promoting activities (Nilausen, 1978). Therefore, the effect of SPS on colony formation by MM200 cells grown in semi-solid agar medium containing either heat-inactivated or heat-inactivated and delipidated FCS was tested. The results of this experiment are shown in Table VIII. It can be seen that delipidated FCS was extremely poor in its ability to support clonal growth and that the lipid in REL did not compensate for the loss of FCS-associated lipid. What is remarkable was the degree to which SPS was able to compensate for this deficiency. Whereas with delipidated serum in the absence of SPS, CEs of 0% and 2±1% were obtained, the addition of SPS to 1 mg ml$^{-1}$ increased the CEs to 47±6% and 52±6%, values that were higher even than the CEs observed with non-delipidated serum in the absence of SPS. Only non-delipidated serum with non-delipidated lysate in the presence of 1 mg ml$^{-1}$ SPS gave a better result, a CE of 65±8%. Thus it would appear that SPS can very effectively compensate for the lack of a growth promoting lipid component in FCS. Repeat experiments involving both MM96 and MM200 cells yielded similar findings.

*$^{125}$I-SPS binding and uptake studies*

$^{125}$I-labelled SPS was used to examine the nature of the interaction of SPS with cells. Studies employing mastocytoma P-815 X-2 showed that, after 15 min exposure at 4°C, $^{125}$I-SPS was bound equally well to both untreated and proteolytic enzyme treated cells. Cells killed by repeated freezing and thawing were several fold less effective in binding $^{125}$I-SPS.

A comparison of MM96 cells that had been incubated with $^{125}$I-SPS for 5 min at 4°C then sorted into $G_1$ and $G_2$ pools on the basis of Hoechst 33342 fluorescence indicated that binding was increased in the case of $G_2$ cells in proportion to their increased surface area over $G_1$ cells.

Other studies showed that the initial binding of $^{125}$I-SPS to MM96 cells was similarly rapid at 4°C and 37°C. However, continued incubation showed further binding to be temperature dependent. Cultures incubated for 24 h at 37°C increased their $^{125}$I-SPS binding 7-fold which when corrected for proliferation corresponded to a 4-fold increase in binding per cell. By contrast those incubated at 4°C remained near constant in their degree of binding (Figure 2).

Studies were also made of the amount of $^{125}$I-SPS bound by MM96 cells after 24 h incubation at either 4°C or 37°C in liquid culture medium. Cells from such cultures were washed 1 to 4 times and then the ratio of $^{125}$I-SPS to residual cell number determined. In addition to confirming the temperature dependent difference in $^{125}$I-SPS labelling at 24 h, it was found that cells incubated at 37°C in REL containing medium bound ~30% less $^{125}$I-SPS than those incubated at 37°C in the absence of REL. Although temperature had a major effect on the amount of $^{125}$I-SPS bound to cells over a 24 h period it was found that similar amounts of

**Table VIII** Effect of SPS on the cloning efficiency of MM200 human melanoma cells cultured in agar medium containing various combinations of non-delipidated and delipidated FCS and REL[a,b,c,d]

| | | *SPS concentration (mg ml$^{-1}$)* | | | | |
|---|---|---|---|---|---|---|
| *FCS treatment* | *REL treatment* | *1.0* | *0.2* | *0.04* | *0.008* | *0* |
| non-delipidated | non-delipidated | 65±8 | 37±4 | 28±6 | 34±9 | 32±6 |
| non-delipidated | delipidated | 51±14 | 51±7 | 48±3 | 34±4 | 42±8 |
| delipidated | non-delipidated | 47±6 | 22±11 | 11±9 | 2±1 | 0 |
| delipidated | delipidated | 52±6 | 20±9 | 21±1 | 15±2 | 2±1 |

[a]Heat-inactivated FCS was used in this study.
[b]Triplicate cultures were used for each experimental condition.
[c]Colonies were scored at 14 days.
[d]Results expressed as % cloning efficiency ± s.d.

**Figure 2** Effect of temperature on the continued binding of $^{125}$I-SPS to MM96 cells, the DNA of which had previously been labelled with $^{14}$C-TdR. Cells incubated in REL-free liquid medium at 37°C (○); cells incubated in REL-free liquid medium at 4°C (●). Bars indicate s.d. Quadruplicate cultures per experimental condition were harvested at each time point.

label were rinsed from both the 4°C and 37°C pre-incubated cells, the 4°C pre-incubated cells retaining little residual label, the 37°C pre-incubated cells retaining considerable label. These results indicated that SPS was bound by cells in both an easy and a difficult (or impossible) to remove form. Repeat experiments yielded similar results.

Autoradiography of washed monolayer cultured MM96 cells that had been incubated at 37°C for 24 h in $^{125}$I-SPS containing REL-free liquid medium indicated that the bulk of the residual label was localised to the cytoplasm. Significant nuclear labelling was not seen (Figure 3). Labelling, although more abundant in vacuolated cytoplasm, could not be localised to any particular intracytoplasmic organelle. Granular material, similar to that described below in the colonies, was also observed in the cytoplasm of these cells but in smaller, less compact masses. Labelling was rarely associated with this material.

*Ultrastructural studies*

Electron microscopy showed that the majority of cells in MM96 colonies growing in agar medium containing 1 mg ml$^{-1}$ SPS with REL had compact masses of granular material in their cytoplasm (Figure 4). These granular masses were not observed in colonies growing in medium with REL alone or neither additive. No other morphological differences were noted.

### Discussion

In 1971 Bradley *et al.* described the enhancement of granulocyte-monocyte colony formation by both intact and lysed red blood cells. Studies on the nature of the enhancing activity indicated it to be associated with the haemoglobin molecule, sulphydryl groups being essential to its activity (Kriegler *et al.*, 1981).

The present study showed that in the presence of REL, SPS was able to further enhance colony formation by a number of anchorage-independent cell lines. Anchorage-independent human melanoma cell lines were found to be particularly responsive to the dual effects of REL and SPS.

Except when low concentrations of SPS were used, the presence of a source of REL was essential to the potentiation of colony formation by SPS. Without REL, SPS was generally found to be highly toxic. The failure of sulphonated resin to remove the detoxifying component in REL suggested that detoxification was not mediated through the masking of sulphonate groups on SPS, delipidation experiments showed that protection was not afforded by a lipid component in lysate and the failure of glutathione to substitute for REL indicated that sulphydryl groups were not involved. The trypsin sensitivity of the component of REL that protected against the toxicity of SPS showed it to be both peptide in nature and to be different from the enhancing activity associated with haemoglobin (Bertoncello & Bradley, 1977; Kriegler *et al.*, 1981).

Colony growth rate studies suggested that SPS exerted its effect by reducing the initial lag prior to colony initiation. REL alone was found to have a similar though lesser effect on lag period reduction. This latter finding was consistent with observations made by Chen & Lin (1981) regarding the effects of mouse red cell lysates on colony formation by mouse macrophage precursors.

Of particular interest was the influence an alteration in lag period had upon colony number,

**Figure 3** Electron microscopic autoradiograph of monolayer cultured MM96 cells that had been incubated at 37°C for 24h in REL-free $^{125}$I-SPS containing liquid medium. Note that the bulk of the residual label was localised to the cytoplasm ×2,500.

**Figure 4** Electron micrograph of two cells in a MM96 colony, growing in agar medium containing 1 mg ml$^{-1}$ SPS with REL, showing compact masses of granular material (arrows) in their cytoplasm ×10,500. *Inset* A higher magnification electron micrograph of such a granular mass ×28,000.

particularly when colonies were scored early. Apart from being important in relation to understanding the actions of SPS and lysate, these findings emphasise the importance of selecting the correct time for scoring colonies Ablett *et al.* (1984) have shown the need to consider the growth rates of primary human tumour-derived colonies when selecting the appropriate time to score such colonies. The decision as when to score would assume even greater importance if, in addition to the intrinsically slow growth rate of such colonies, cytotoxic regimens were to have an opposite effect on lag to that of SPS and REL in causing a prolongation of this phase. If such is the case then the early scoring of colony numbers could give a very misleading indication of drug sensitivity.

Studies into the mechanisms by which SPS influenced culture conditions indicated that its effectiveness varied according to the species origin of the serum used. Studies also showed cultures containing poor batches of FCS were improved far more in their colony growth supporting ability than cultures containing sera obtained from superior batches. These results were consistent with SPS exerting its potentiating effect by negating the effect of a growth inhibitor and/or compensating for a deficiency in the quantity of a growth promoting component.

Further investigations involving colony formation by GM-CFC in both heat-inactivated and uninactivated FCS containing medium were consistent with the hypothesis that one action of SPS was to interfere with the expression of a thermolabile growth inhibitor. However, the observation that MM96 cells formed colonies equally well in medium containing uninactivated serum as heat-inactivated serum, and that SPS potentiated colony formation equally well in medium containing either type of serum, suggests that the major way by which SPS exerted its potentiating influence was not by interferring with the expression of a serum inhibitor. Subsequent studies in which either growth inhibiting very-low-density-lipoprotein-rich BALB/c serum (Metcalf & Russell, 1976) or delipidated BALB/c serum were used showed that SPS effectively protected lipoprotein-inhibitor-sensitive cells from the action of these inhibitors. Because of the marked clearing effect of SPS on lipoprotein-rich serum containing agar medium it was speculated that the inhibition reducing property of SPS may have been mediated by the detergent action of SPS (Eng, 1975). The failure to confirm this hypothesis with other detergents, which however also failed to reduce the turbidity of the medium, may have been due to their inadequate activity when tested at non-toxic concentrations.

In addition to blocking the effects of serum inhibitory factors, SPS had a direct positive effect on colony formation. This was shown by studies in which delipidated FCS was substituted for non-delipidated FCS. In these studies it was found that SPS had the remarkable property of more than compensating for the loss of an otherwise important lipid growth-promoting activity in FCS.

Finally it was shown that $^{125}$I-SPS bound rapidly but loosely in a temperature independent manner to viable cells. Neither the previous proteolytic enzyme treatment of the cells nor the concommitant presence of REL affected this binding. Both $G_1$ and $G_2$ cells bound $^{125}$I-SPS, binding being proportional to the surface area of the cells. Continued incubation at 37°C but not 4°C, caused further accumulation of label, uptake being somewhat less in the presence of REL. The decrease in active binding of $^{125}$I-SPS in the presence of REL was far too small to account for the dramatic reduction in SPS toxicity observed in the presence of REL. Autoradiography revealed the intracytoplasmic location of $^{125}$I-SPS in cells that had been incubated for 24 h at 37°C. Presumably the internalised $^{125}$I-SPS accounted for the label that could not be washed from cells that had been incubated at 37°C. Labelling was abundant in vacuolated cytoplasm with no association apparent between such label and the ultrastructurally observed intracytoplasmic granular masses. It remains unknown whether it is the binding by SPS to low affinity cell receptors that enables cells to compensate for the lack of an otherwise important growth promoting serum lipid.

The above study was confined to the effects of SPS on normal cells that had been freshly obtained, and transformed cells with a prolonged history of *in vitro* culture. Investigations are now commencing into the effects of SPS on colony formation by freshly dissociated tumour cells. These studies will be reported separately.

This work was supported by the National Health and Medical Research Council of Australia and the Queensland Cancer Fund. We are also grateful to the Queensland Cancer Fund for providing us with access to the fluorescence activated cell sorter.

### References

ABLETT, G., BISHOP, C., SHERIDAN, J.W. & DONALD, K.J. (1978). Inhibition of growth of murine tumour cells *in vitro* by serum from non-immune syngeneic and allogeneic mice. *Br. J. Exp. Pathol.*, **59**, 552.

ABLETT, G.A., SMITH, P.J., SHERIDAN, J.W. & LIHOU, M.G. (1984). Limitations of the agar colony-forming assay for the assessment of paediatric tumours. *Br. J. Cancer*, **50**, 000.

ALLGÖWER, V.M. (1947). Ueber die wirkung von heparin, polanetholsulfosaurem natrium (Liquoid Roche) und tribasischem natriumcitrat auf menschliche leukozyten *in vitro*. *Schweiz. Med. Wochenschr.*, **77**, 40.

BALL, A.K., TIDBALL, J.G. & DICKSON, D.H. (1981). An alternative to the flat substrate method of preparing electron microscope autoradiographs. *Stain Technol.*, **56**, 239.

BERTONCELLO, I. & BRADLEY, T.R. (1977). The physiochemical properties of erythrocyte derived activity which enhances murine bone marrow colony growth in agar culture. *Aust. J. Exp. Biol. Med. Sci.*, **55**, 281.

BRADLEY, T.R., TELFER, P.A. & FRY, P. (1971). The effect of erythrocytes on mouse bone marrow colony development *in vitro*. *Blood*, **38**, 353.

CHAM, B.E. & KNOWLES, B.R. (1976). A solvent system for delipidation of plasma or serum without protein precipitation. *J. Lipid Res.*, **17**, 176.

CHAN, S.H. (1971). Influence of serum inhibitors on colony development *in vitro* by bone marrow cells. *Aust. J. Exp. Biol. Med. Sci.*, **49**, 553.

CHEN, D.-M. & LIN, H.-S. (1981). Differential enhancement of the clonal growth of various mononuclear phagocytes by hemolysates. *J. Reticuloendothel. Soc.*, **29**, 465.

ENG, J. (1975). Effect of sodium polyanethol sulfonate in blood cultures. *J. Clin. Microbiol.*, **1**, 119.

KRIEGLER, A.B., BRADLEY, T.R., HODGSON, G.S. & MCNIECE, I.K. (1981). Identification of the "factor" in erythrocyte lysates which enhances colony growth in agar cultures. *Exp. Hematol.*, **9**, 11.

MÅRDH, P.-A. (1975). Elimination of mycoplasmas from cell cultures with sodium polyanethol sulphonate. *Nature*, **254**, 515.

METCALF, D. (1976). Role of mercaptoethanol and endotoxin in stimulating B lymphocyte colony formation *in vitro*. *J. Immunol.*, **116**, 635.

METCALF, D. & RUSSELL, S. (1976). Inhibition by mouse serum of haemopoietic colony formation *in vitro*. *Exp. Hematol.*, **4**, 339.

NILAUSEN, K. (1978). Role of fatty acids in growth-promoting effect of serum albumin on hamster cells *in vitro*. *J. Cell. Physiol.*, **96**, 1.

SHERIDAN, J.W., BISHOP, C.J. & SIMMONDS, R.J. (1981). Biophysical and morphological correlates of kinetic change and death in a starved human melanoma cell line. *J. Cell Sci.*, **49**, 119.

SHERIDAN, J.W. & METCALF, D. (1973). A low molecular weight factor in lung-conditioned medium stimulating granulocyte and monocyte colony formation *in vitro*. *J. Cell Physiol.*, **81**, 11.

SHERIDAN, J.W., & SIMMONS, R.J. (1981). Studies on a human melanoma cell line: effect of cell crowding and nutrient depletion on the biophysical and kinetic characteristics of the cells. *J. Cell Physiol.*, **107**, 85.

SHERIDAN, J.W. & SIMMONS, R.J. (1983). Pancreatin-EDTA treatment affects buoyancy of cells in Cohn fraction V protein density gradients without residual effect on cell size. *Aust. J. Exp. Biol. Med. Sci.*, **61**, 727.

TAYLOR, I.W. & MILTHORPE, B.K. (1980). An evaluation of DNA fluorochromes, staining techniques and analysis for flow cytometry. *J. Histochem. Cytochem.*, **28**, 1224.